# Supplementary material for: Evaluation of the Pragmatic Implementation of a Digital Health Intervention Promoting Healthy Nutrition, Physical Activity, and Gestational Weight Gain for Women Entering Pregnancy at a High Body Mass Index
Source: Nutrients. 2023 Jan 22;15(3):588. doi: 10.3390/nu15030588 (PMC9921852; doi:10.3390/nu15030588)
Supplement: Supplementary file 1 [file nutrients-15-00588-s001.zip › nutrients-2143997-supplementary.pdf]

Supplement Table S1: Program implementation: participant feedback regarding the txt4two program

Variable	Element	Score and comments, where appropriate
Overall program	Rating (n =14)	*Extremely helpful 35.7%; Moderately helpful 50.0%; A little helpful 7.1%; Missing 7.1% (n=1)*
	Recommend to a friend?	*Yes 64.3%; Maybe 28.6%; Missing 7.1%*
	Liked most (n = 10)	*1 x Being aware of weight gain*<br>*2 x accountable*<br>*Being made accountable for my nutrition and working towards a goal for the pregnancy - I'm a very competitive person; It keeps you on track, accountable, and provides important reminders)*<br>*2 x the goal setting process*<br>*(The initial appointments and goal setting; The initial consult with the dietitian was very helpful in problem solving a couple of things I found hard at that time (especially not having time to eat lunch at work then eating something unhealthy on the way home) I took her advice on directly and bought a few 'convenience' items each week for lunch that I never would have considered as I always make lunches from scratch (but wasn't doing so due to time) meaning I ended up eating a nutrient dense lunch instead of an afternoon tea each day which was a very positive change)*<br>*6 x Reminders and encouragement towards goals/the goal setting session/ friendly and encouraging*<br>*(Good links to info and reminders to keep with my goals; It keeps you on track, accountable, and provides important reminders; It was a nice reminder to keep exercising; Reminders to eat healthily; The encouragement and positive messaging; The staff were friendly and the texts were very encouraging even when I didn't do the right thing)*<br>*1 x links in the texts to physio type exercises (pelvic floor etc).*
	Liked least (n = 7)	*3 x nil*<br>*1 x need more variety and person specific considerations*<br>*1 x some information was off due to changes to my pregnancy due date*<br>*1 x the goals didn't quite align with initial discussion. I think the dietitian just noted the conversation wrong e.g. the exercise goal was different to what we discussed and I kept getting texts to ask me to set a dietary goal and I would reply saying I was still working on the initial dietary goal>> Bit that didn't make a big impact as I still benefited from the initial discussion. I also do not like some of the old fashioned advice in the booklet about low fat dairy 'diet' products. I guess it makes sense if you eat poor quality foods habitually but if you tend to eat good quality whole foods it doesn't apply. There's no way I'd switch from a real food like butter to margarine or eat crappy diet yoghurt, You shouldn't lump something like a croissant in with cakes etc filled with processed sugar. Some of the texts were just annoying. That's probably more to do with my irritable hormonal state rather than their content!!*<br>*1 x there was no phone call check in later in the program to assess if there were any changes to take into consideration*
	Other recommendations	*1 x online weight tracker*<br>*1 x make it clearer when I should be responding to messages*<br>*4 x maybe another short consult halfway through? Would be great to have more videos/ access to stuff from the physios at the Mater. I know it's available but directly linking it to the program would be great; more than one appointment; more 1:1 appointments; schedule a telehealth call part way through the program to reassess goals, exercise abilities eg I was recovering from hyperemesis but have SPD so diet can improve dramatically but exercise was challenging*<br>*1 x more flexibility to allow changes eg change due dates; but also change goals. It became really repetitive after a while and I changed from walking to swimming for a few weeks there just for something different, but didn't have an easy avenue to change goals. Maybe having a profile that we can access to easily change and update our goals and info would be good. And also so I can track my own progress*

		as well. For example, I'd like to know how many weeks in total did I achieve my foal and how many didn't I and why was that?<br>*1 x more tools and suggestions*<br>*1 x nil*
Booklet	Amount read (n = 14)	*All 64.3%; About half 14.3%; A few pages 14.3% ; Missing 7.1%*
Website	Most liked (n = 8)	*1 x goal planner*<br>*4 x Informative; the amount of information available; useful info and resources*<br>*2 x Quick and simple; simple and easy to navigate*<br>*1 x The accountability check ins*
	Least liked	*5 x n/a*<br>*2 x Needed a more interactive weight monitor*
Texts	Number received	*Too many 21.4% (n=3); Just right 71.4%; Missing 7.1% (n = 1)*
	Timing (n = 14)	*Acceptable 92.9%; Missing 7.1% (n=1)*
	Percentage read (n = 14)	*100% of texts 85.7%; 0% of texts 7.1% (n=1) ; Missing 7.1%*
	Liked most (n =12)	*4 x Concise but helpful; Helpful info; Links to useful info; The handy tips*<br>*9 x reminders; Encouragement; the check in regarding weekly goals; questions about progress, goals and weight tracker; Positive reinforcement of reaching goals; Reminded me to eat better; The daily reminders to keep on track; The reminders and the encouragement through texts*<br>*2 x Interesting facts about the baby and pregnancy; The simple food-related texts were good eg reminder that fatty acids good for baby so eat fish etc*
	Liked least (n = 9)	*4 x nil*<br>*1 x I had changes to my due date and as such, the timing of the weekly texts was off. I didn't know how to resolve this*<br>*1 x my pregnancy situation and the goals changed and the texts were therefore not very relevant*<br>*1 x not interested in standard 'facts' about the baby eg 'hearing is fully developed' – I have an app for that, it was just more crap popping up on my phone. Found some texts annoying just popping up on my phone in this chipper sort of tone when in the hospital and unwell*<br>*1 x wasn't sure if I should reply to some of them or not*<br><br>*1 x I think it would be more helpful for someone less educated or aware of healthy pregnancy and weight management*<br>*1 x Thank you for the guidance and resources provided. This was very helpful and useful to me*
